# Recent Studies and Progress in the Intratumoral Administration of Nano-Sized Drug Delivery Systems

**DOI:** 10.3390/nano13152225

**Published:** 2023-07-31

**Authors:** Wan Su Yun, Jeongrae Kim, Dong-Kwon Lim, Dong-Hwee Kim, Seong Ik Jeon, Kwangmeyung Kim

**Affiliations:** 1 Korea Institute of Science and Technology (KU-KIST), Graduate School of Converging Science and Technology, Korea University, Seoul 02841, Republic of Korea; 2 College of Pharmacy, Graduate School of Pharmaceutical Sciences, Ewha Womans University, Seoul 03760, Republic of Korea

**Keywords:** intratumoral administration, nanomedicine, targeted cancer therapy, localized therapy, drug delivery system

## Abstract

Over the last 30 years, diverse types of nano-sized drug delivery systems (nanoDDSs) have been intensively explored for cancer therapy, exploiting their passive tumor targetability with an enhanced permeability and retention effect. However, their systemic administration has aroused some unavoidable complications, including insufficient tumor-targeting efficiency, side effects due to their undesirable biodistribution, and carrier-associated toxicity. In this review, the recent studies and advancements in intratumoral nanoDDS administration are generally summarized. After identifying the factors to be considered to enhance the therapeutic efficacy of intratumoral nanoDDS administration, the experimental results on the application of intratumoral nanoDDS administration to various types of cancer therapies are discussed. Subsequently, the reports on clinical studies of intratumoral nanoDDS administration are addressed in short. Intratumoral nanoDDS administration is proven with its versatility to enhance the tumor-specific accumulation and retention of therapeutic agents for various therapeutic modalities. Specifically, it can improve the efficacy of therapeutic agents with poor bioavailability by increasing their intratumoral concentration, while minimizing the side effect of highly toxic agents by restricting their delivery to normal tissues. Intratumoral administration of nanoDDS is considered to expand its application area due to its potent ability to improve therapeutic effects and relieve the systemic toxicities of nanoDDSs.

## 1. Introduction

Nano-sized drug delivery systems (nanoDDSs) have become a major strategy for targeted cancer therapy. NanoDDSs promote the extended blood circulation of encapsulated anticancer drugs by preventing their rapid renal clearance and improving their physiological stability [1,2]. In addition, they can be specifically accumulated inside the tumors via the enhanced permeability and retention (EPR) effect, which is attributed to the abnormal tumor vascular morphology and dysfunctional lymphatic system [3,4,5,6]. Based on their unique features, diverse types of nanoDDSs have been explored for the tumor-specific delivery of various anticancer drugs [7,8,9]. Despite the extensive studies on nanoDDS-mediated cancer therapy, however, it is still challenging to overcome the complications associated with the systemic administration of nanoDDSs. Their passive tumor targeting was demonstrated only in animal experiments, not in clinical trials [10,11]. In systemic administration, the impact of nanoDDSs on the actual tumor delivery efficiency is not very significant compared to free drugs, and considerable proportions of them are undesirably distributed to normal tissues [12,13,14]. NanoDDSs often interact with blood components or are severely entrapped by reticuloendothelial systems (RESs) in the liver and spleen during their circulation, eventually being cleared before reaching tumor tissues [15,16]. The nanoDDSs mistakenly delivered to normal tissues cause side effects induced not only by anticancer drugs but also by nanocarriers [17]. Great efforts have been made to improve the delivery efficiency of systemically administered nanoDDSs, such as functionalization with tumor-targeting ligands or tumor stimuli-sensitive moieties, but these have not elicited any dramatic changes yet [18,19,20].

**Scheme 1:**
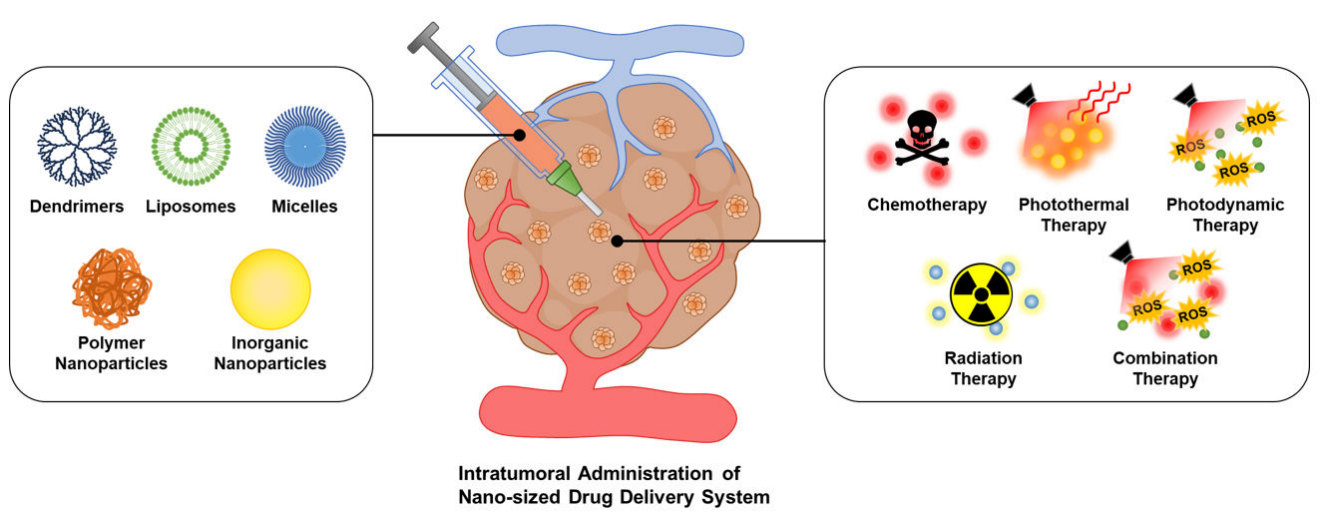
Schematic illustration of intratumoral nanoDDS administration. Various types of nanoDDSs can be administered intratumorally for different therapeutic approaches, enhancing their tumor-specific accumulation and diminishing off-target delivery.

To overcome the limitations of systemic administration, the intratumoral administration of nanoDDSs was proposed as an alternative approach [21,22]. Intratumoral nanoDDS administration has been increasingly studied within the last decade (2013–2023), with approximately 2300 reports, 1.5-fold more than in the previous decade (2002–2012), due to its outstanding biodistribution control and superior therapeutic outcomes. Since nanoDDSs are directly injected into tumor tissues, it is possible to evade their entrapment by RESs and maximize their tumor accumulation [23,24]. Moreover, differently from free drug injection, the utilization of nanoDDSs for intratumoral administration can prevent the fast dissipation and washout of drugs from tumor tissues to blood streams and surrounding normal tissues by controlling their diffusivity [25]. The intratumoral administration of nanoDDSs can ensure enhanced therapeutic outcomes and mitigated off-target toxicity in various therapeutic modalities compared to their systemic administration (Scheme 1). Various types of nanoDDSs, including liposomes, micelles, polymeric nanoparticles, nanogels, and inorganic nanoparticles with different physicochemical properties, have been exploited for their intratumoral administration and exhibited different therapeutic outcomes. Herein, a broad review of the current studies and progress in the intratumoral administration of nanoDDSs and their therapeutic outcomes is provided, mainly focused on the advancements within the last 10 years (2012–2023). First, the factors affecting the intratumoral administration efficiency of nanoDDSs are classified as tumor microenvironment (TME)-, nanoDDS-physicochemical-property-, and administration-method-associated ones, and their most desirable conditions are briefly addressed. The advantages of applying the intratumoral administration of nanoDDSs depending on the treatment methods are subsequently explained, and the actual research cases and their therapeutic outcomes are summarized. Finally, recent advances in the clinical trials of intratumoral nanoDDS administration are noted.

## 2. Factors Regulating the Therapeutic Efficacy of Intratumoral Administration

Obviously, the intratumoral administration of nanoDDSs could enhance their tumor-specific accumulation and alleviate their undesirable distribution to normal tissues, which has been fully demonstrated in several experimental results. According to the study by C. Santini et al., intratumorally administered Pluronic P94 copolymer nanoparticles exhibited a 13.1-fold higher intratumoral level than intravenously administered ones at 48 h after injection [26]. Most of the intravenously administered nanoparticles were found in the liver, whereas those with intratumoral administration were delivered to the liver at a 2.1-fold lower level. Moreover, the utilization of nanoDDSs for intratumoral administration assists in the achievement of better therapeutic outcomes compared to small molecular drugs by prolonging the intratumoral retention time. Due to their excessive diffusivity, small molecular drugs are often rapidly washed out from the injected tumor tissues, reducing their therapeutic efficacy and causing systemic toxicities. NanoDDSs can regulate the diffusivity of encapsulated drugs and diminish their tumor escape, leading them to affect tumor tissues exclusively. H. Xie et al. examined the biodistribution of ^64^Cu-DOTAs and ^64^Cu-labeled gold nanoshells (~140 nm in diameter) depending on their formulations and administration routes, determining that the amount of intratumorally administered gold nanoshells retained inside the tumor tissue at 46 h post-injection was 8.2-fold higher than that of intravenously administered ones [27]. It is worth noting that the tumor-remaining percentage of intratumorally administered ^64^Cu-labeled gold nanoshells was also 3.8-fold higher than that of ^64^Cu-DOTA, which is the small molecular form of the label, demonstrating the enhanced retention effect of nanoDDSs inside tumor tissues. The improved tumor accumulation of nanoDDSs due to their intratumoral administration is directly accompanied by their increased therapeutic efficacy. When curcumin-loaded poly(lactide-co-glycolide) (PLGA) nanoparticles were intratumorally administered to RG2 glioma-bearing rats, they successfully reduced the size of tumor tissue by 52% from the initial size, while intravenously administered tumors grew by 252% after 5 days [28]. The study by Y. Jin et al. revealed the superior antitumor efficacy of photothermal therapy (PTT) using intratumorally administered tantalum oxide-encapsulated polypyrrole nanoparticles (TaOx@PPys) as well, wherein the tumor growth inhibition rates of PTTs with intratumoral and intravenous administration of TaOx@PPys were 100% (complete remission) and 66.5%, respectively [29].

**Scheme 2:**
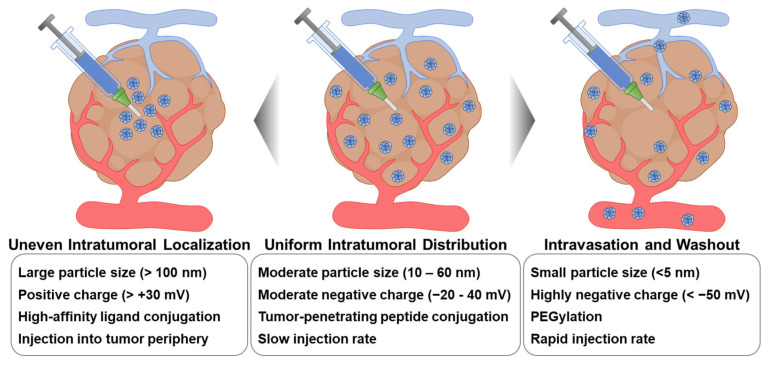
External factors affecting the intratumoral distribution of intratumorally administered nanoDDSs. To achieve maximal therapeutic outcomes, intratumorally administered nanoDDSs should be uniformly distributed throughout the tumor tissue without any intravasation or washout. Therefore, external factors affecting the intratumoral distribution of nanoDDSs, such as particle size, surface charge and functionalization, and injection site and rate, should be precisely regulated.

Although the intratumoral administration of nanoDDSs basically ensures much improved therapeutic outcomes compared to systemic administration, several things should be considered to increase their therapeutic efficacy. To draw out the maximal therapeutic efficacy of nanoDDSs while diminishing their side effects through their intratumoral administration, (i) the administered nanoDDSs should be uniformly dispersed throughout the entire tumor tissue, (ii) they should remain for a prolonged time without leaking out from the tumor tissues, and (iii) they should be actively taken up by cancer cells before their escape. There are several internal and external factors determining the intratumoral dispersion and washout of intratumorally administered nanoDDSs, such as particle size and surface property, TMEs, and injection site and rate, which are described in detail below (Scheme 2).

### 2.1. Tumor Microenvironments

Tumor tissues elicit uncommon cellular microenvironments distinguishable from those of normal tissues, which can affect the intratumoral diffusion and localization of nanoDDSs. Highly permeable abnormal blood vessels are irregularly organized surrounding the tumor periphery, whereas lymphatic drainage is not sufficiently developed throughout the tumor tissues [30,31]. The leaky peripheral vasculature and lymphatic dysfunction of tumor tissues may facilitate the passive tumor targeting of systemically delivered nanoDDSs via the EPR effect, but on the other hand, they cause high intratumoral interstitial fluid pressure (IFP) and disturb the deep penetration of nanoDDSs [32,33,34]. Attributed to interstitial fluids exuded out from leaky blood vessels and hardly drained out from tumor tissues, the intratumoral IFP is 2–20-fold higher than that of normal tissues [35,36]. Tumor tissues of larger volumes tend to have higher IFP, and the prevalent expression of angiogenic factors such as vascular endothelial growth factor (VEGF), platelet-derived growth factor (PDGF), and transforming growth factor-β (TGF-β) also increase the intratumoral IFP [37,38,39,40]. Intratumoral IFP is one of the major obstacles to intratumoral nanoDDS delivery since it causes abnormal convective flow inside the tumor tissue and impairs the penetration of nanoDDSs to the tumor core. When nanoDDSs are intratumorally administered into the marginal region of tumor tissues, the high outward IFP of tumors would hinder their uniform intratumoral dispersion and force them to be washed out from tumor tissues [41,42,43].

The stiff extracellular matrix (ECM) and high stromal density of tumor tissues are other hurdles for the intratumoral distribution of nanoDDSs. Compared to the normal ECM, the tumoral ECM contains a higher level of collagen type I which is produced by cancer-associated fibroblasts (CAFs), and the excessive collagen level significantly strengthens the tumoral ECM tension [44,45,46,47]. Several growth factors and enzymes overexpressed inside tumor tissues are also involved in the enhancement of tumoral ECM stiffness, wherein fibroblast activation protein α (FAP) and interleukin-1 and -6 (IL-1 and -6) activate the collagen synthesis by CAFs, and lysyl oxidase (LOX) is responsible for the chemical crosslinking of collagen [48,49,50,51]. In addition to the stiff tumoral ECM, the tumor stroma is compactly filled with recruited stromal cells, including CAFs, tumor-associated macrophages (TAMs), and regulatory T cells (Tregs), which further narrows the diffusion path of nanoDDSs [52,53,54]. In particular, TAMs additionally obstruct the intratumoral dispersion of nanoDDSs by their phagocytosis [55]. Furthermore, the core of solid tumors is densely packed with necrotic cells due to the lack of blood vessels and consequent severe regional hypoxia, which is difficult for nanoDDSs to penetrate through [56,57]. The stiff ECM and dense stroma of tumor tissues severely restrict both the convective flow and diffusion of intratumorally administered nanoDDSs, leading to their uneven localization inside tumor tissues [54,58,59].

### 2.2. NanoDDS Properties

The physicochemical properties of nanoDDSs are closely related to their intratumoral distribution as they determine the interaction between nanoDDSs and compartments and diffusivity through the tumor interstitium. The first consideration for efficient intratumoral administration is particle size. It is known that the size of nanoDDSs should be precisely controlled for their systemic administration since it greatly regulates their biodistribution and tumor accumulation. The most desirable nanoDDS size range was reported to be 5–100 nm for effective passive tumor targeting without renal clearance or entrapment by RESs [7,43]. NanoDDSs over 200 nm are hardly delivered to tumor tissues via the EPR effect and are predominantly distributed in the liver and spleen [60,61]. On the contrary, intratumoral administration is applicable to more various sizes of nanoDDSs compared to systemic administration since it does not require the blood circulation and tumor targeting of nanoDDSs. Even micro-sized particles can be administered intratumorally on demand, which is not available in systemic administration due to their poor stability during circulation [62,63,64]. However, to obtain better therapeutic outcomes, the size of nanoDDSs should be controlled to an appropriate range for their diffusion and uniform distribution within the tumor. NanoDDSs with smaller sizes tend to more freely diffuse through the tumor stroma than larger particles. M. R. Dreher et al. evaluated the intratumoral penetration of dextrans with different molecular weights from 3.3 kDa to 2 MDa, discovering that 3.3 kDa dextran (3.5 nm) exhibited the fastest intratumoral diffusion [65]. Nevertheless, 3.3 kDa dextran was not suitable for intratumoral delivery because it promptly washed out from tumor tissues with its excessive diffusivity. In addition, 2 MDa dextran was not sufficiently accumulated inside tumor tissues either due to its poor penetration ability, and dextrans with molecular weights of 40 and 70 kDa (5–7 nm) showed the most desirable intratumoral accumulation and distribution. In the study by Z. Popovic et al., the intratumoral distribution of differently sized silica quantum dots (12, 60, and 125 nm) was assessed, wherein the 12 nm quantum dot showed the most smooth intratumoral diffusion and uniform distribution, while 60 and 120 nm quantum dots rarely penetrated deep tumor tissues [66]. T. T. Goodman et al. predicted the effect of particle size on the intratumoral diffusion, which was also investigated using polystyrene nanoparticles, and concluded that 20–40 nm particles would effectively diffuse into deep tumor tissues, whereas 100–200 nm particles would not [67]. In addition, the size of nanoDDSs affects their cellular uptake profile [68]. Since cancer cells uptake nanoparticles via the pinocytosis and caveolae- or clathrin-mediated endocytosis pathway, smaller particles are more actively taken up by cancer cells [69,70]. Considering the interstitial diffusion, intravasation, and endocytosis of nanoDDSs, their most appropriate size for intratumoral administration is determined to be 10–60 nm [71].

Secondly, the morphology and surface charge of nanoDDSs should be considered for their efficient intratumoral administration. V. P. Chauhan et al. investigated the tumor interstitial transport of quantum-dot-encapsulated nanospheres (~35 nm) and nanorods (~54 nm length and ~15 nm diameter) which have similar hydrodynamic sizes, confirming that the volume of nanorods distributed deep inside the tumor tissue was 1.7-fold higher than that of nanosphere [72]. K. C. L. Black et al. also assessed the intratumoral distribution of ^198^Au-doped nanospheres, nanodisks, nanorods, and nanocages with similar sizes of ~50 nm [73]. It was discovered that the nanorods and nanocages could penetrate the center of tumor tissues, while nanospheres and nanodisks predominantly remained in the marginal tumor region. The morphology of nanostructures is considered to readjust their convective motion and diffusivity inside tumor tissues and thereby change their intratumoral distribution. The surface charges of nanoDDSs are associated with their interaction with cancer cells or TMEs and their cellular internalization. Due to the strong interaction with the negatively charged cell membrane, nanoparticles with positive surface charges are known to be more actively taken up by cancer cells than those with neutral or negative charges [74,75]. However, their strong interaction with cancer cells and vigorous cellular uptake are not advantageous for their intratumoral dispersion since they would be restrictively accumulated in the right region of administration. In the study by R. B. Campbell et al., cationic liposomes (~150 nm diameter and +31 mV surface charge) were mostly accumulated around the tumor vessels rather than being dispersed into the deep tissue [76]. A study to compare the intratumoral distribution of positively (+30 mV) and negatively (−36 mV) charged gold nanoparticles (6 nm) was performed as well, wherein the negatively charged nanoparticles exhibited faster intratumoral diffusion and more efficient delivery to tumor cores [77]. Furthermore, D. L. Priwitaningrum et al. demonstrated that silica nanoparticles (30 nm) with higher negative charges (−40 mV) could more deeply diffuse into the core of the tumor spheroid compared to those with moderate charges (−23 mV) [78]. However, it is necessary to control the surface charge of nanoDDSs precisely since the excessive negative charges hinder their endocytosis.

The surface modification of nanoDDSs is an attractive method to modulate their intratumoral distribution since it can easily endow nanoDDSs with additional functionalities. One of the most commonly performed modifications is surface PEGylation, frequently used to prolong the blood half-life of nanoDDSs [79,80,81]. The antifouling effect of PEG improves their physiological stability by preventing the surface adhesion of biological components [82]. In addition, PEGylated nanoDDSs were proven to be more smoothly diffused through the tumor interstitium with reduced interaction with cancer cells and tumor stroma [83]. Nevertheless, the length of PEG and PEGylation density should be carefully controlled due to the adverse effect of PEGylation on nanoDDS endocytosis [84,85]. Another candidate for the surface modification of nanoDDSs are active targeting ligands. The targeting ligands on the nanoDDS surface not only facilitate the endocytosis of nanoDDSs but also assist with their retention inside tumor tissues without their intravasation and washout [86]. N. Chattopadhyay et al. compared the tumor retention of bare and trastuzumab-antibody-conjugated gold nanoparticles after their intratumoral administration [87]. Due to the HER-2 binding effect of trastuzumab, a 1.3-fold larger amount of antibody-conjugated gold nanoparticles remained inside the tumor tissue than the bare nanoparticles 48 h post-administration. In another study by J. Lin et al. mPEG-PLA nanospheres with or without the folate-receptor-binding methotrexate (MTX) conjugation were prepared, and their therapeutic effects after intratumoral administration were evaluated [88]. The MTX-conjugated nanospheres showed much-prolonged tumor retention for 48 h compared to those without MTX, which was accompanied by improved tumor growth inhibition. It is worth noting that the use of targeting ligands with strong affinity would impair the intratumoral diffusion of nanoDDSs and cause their heterogeneous localization [71,86]. Surface modification with tumor-penetrating peptides (TPPs) is a favorable strategy that has been broadly conducted for the deep tumor penetration of nanoDDSs. TPPs utilize the trans-tissue transport pathway of cancer cells to reach the central region of tumor tissues [89,90]. The application of TPPs to intratumorally administered nanoDDSs can facilitate their uniform intratumoral distribution. R. Chen et al. administered the recombinant protein containing proapoptotic peptides and TPPs intratumorally and observed its intratumoral spreading and therapeutic effect [91]. The protein with the TPP domain could be more evenly dispersed throughout the tumor tissues than that without the TPP, showing a 77% higher antitumor efficacy. However, the severe toxicity of TPP is a significant hurdle for its application in intratumoral administration [92].

### 2.3. Administration Process

The intratumoral administration process of nanoDDSs, including injection dose, rate, and site, significantly affects their therapeutic outcomes. Although the physicochemical properties of nanoparticles are optimally adjusted, their efficacy will be drastically diminished without an adequate intratumoral administration process. Considering the outward IFP, nanoDDSs should be injected near the center of the tumor tissue. The nanoDDSs administered in the tumor periphery hardly diffuse into the tumor core due to the high IFP, and then, a significant proportion of them will be rapidly washed out. The multiple-point injection of nanoDDSs is a useful approach for their uniform intratumoral distribution [93,94]. The dose and injection rate should be also specifically controlled accounting for the size of tumor tissues. The appropriate dose for intratumoral administration is far lower than that for systemic delivery, considering the difference in tumor accumulation efficiency between them. If the nanoDDS dose is too high or the injection rate is too fast compared to the tumor size, the excess will leak out and cause off-target toxicity. Conversely, nanoDDSs will not be sufficiently administered due to the tumor IFP if their injection rate is too slow.

In clinical trials, except for in skin cancers, the intratumoral administration of drugs unavoidably requires the use of long needles or open surgery due to the limited accessibility of deep tumor tissues, which is the most critical limitation in its application [95,96]. The intratumoral administration of drugs through long needles can injure other normal organs, and injection with open surgery is highly invasive. Therefore, intratumoral administration is often carried out after the resection of solid tumors by infusing drugs into the resection cavity through a catheter to treat the possible remaining tumor tissues [97,98]. However, this administration method makes it difficult to predict the intratumoral dispersion of administered drugs and their undesirable delivery to normal tissues. Recently, the utilization of interventional devices has been investigated for intratumoral drug administration [99,100,101]. Interventional therapy is a minimally invasive intratumoral drug administration method wherein drugs are intratumorally administered through a microcatheter navigated to tumor tissues along the blood vessels. Through interventional device-guided intratumoral administration, it is possible to precisely control the exact injection sites, drug doses, and injection rates. However, the tumor sites where the microcatheter can reach are restricted, and a non-invasive real-time visualization technique with high resolution is necessary to identify the specific site of administration.

## 3. Intratumoral-Administration-Mediated Therapeutic Approaches

In preclinical research, the intratumoral administration of nanoDDSs has been widely employed in various therapeutic modalities, including chemotherapy, photothermal therapy (PTT), photodynamic therapy (PDT), and radiation therapy, demonstrating superior therapeutic outcomes compared to their systemic administration [102]. In addition, intratumoral nanoDDS administration broadens its application areas to cancer immunotherapy. This chapter provides a detailed account of the application of the intratumoral administration of nanoDDS in each therapeutic modality and presents the therapeutic outcomes. The studies that applied intratumoral nanoDDS administration to different therapeutic modalities are briefly summarized in Table 1.

### 3.1. Chemotherapy

Chemotherapy is a basic and traditional approach to cancer treatment, which still suffers from serious side effects in its systemic administration. Therefore, the application of intratumoral nanoDDSs to chemotherapy would be remarkably beneficial in alleviating its side effects. Various methods are employed for the intratumoral administration of nanoDDSs for chemotherapy, including syringe-guided injection, hydrogel-based delivery, and microneedle-based delivery [121,122,123,124,125]. Syringe-guided injection is the most common approach entailing the direct intratumoral administration of drug-loaded nanoparticles. This method offers simplicity and intuitiveness, providing the advantage of direct tumor delivery without the need for additional compounds. Moreover, it enables the attainment of high drug concentrations within the tumor due to enhanced targeting efficiency. J. Kim et al. reported the intratumoral administration of doxorubicin-encapsulated biodegradable PLGA nanoparticles [103]. The nanoparticles exhibited a sustained doxorubicin release profile, about 46.6% for 14 days, indicating its potential for a controlled drug delivery vehicle. The PLGA nanoparticles were confirmed with their property to induce apoptotic cell death in vitro. Subsequent in vivo evaluation using the Balb/c mouse models revealed that the intratumorally administered PLGA nanoparticles not only promoted a significant regression in tumor growth but also stimulated the immune response against the tumor, highlighting their capacity to exert antitumor effects. Importantly, intratumoral administration of the nanoparticles was demonstrated to have an enhanced therapeutic efficacy with their sustained drug release behavior, resulting in reduced tumor size in their following repeated administrations.

**Figure 1:**
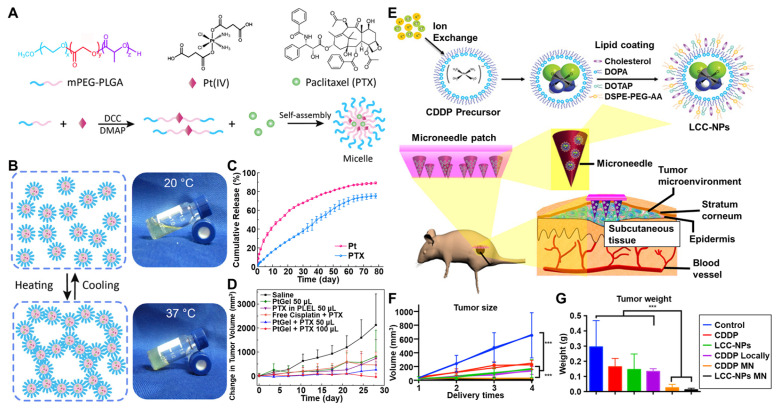
Intratumoral-nanoDDS-administration-mediated chemotherapy. (**A**) Thermosensitive polymeric micelles loaded with both cisplatin and paclitaxel were prepared for intratumorally administered chemotherapy. (**B**) Thermosensitive polymeric micelles underwent a phase transition to a gel state. (**C**) The cumulative release profiles of cisplatin and paclitaxel were observed. (**D**) The tumor size changes after intratumoral administration of polymeric micelles were measured over time. Reproduced with permission [104]. Copyright 2017, ACS Publications. (**E**) The microneedle-mediated intratumoral administration of cisplatin-encapsulated nanoparticles was proposed. (**F**) The sizes and (**G**) weights of tumors with microneedle-mediated administration were significantly regressed compared to those with intravenous and instant intratumoral administrations. *** *p* < 0.05. Reproduced with permission [106]. Copyright 2018, ACS Publications.

Nevertheless, syringe-guided intratumoral nanoDDS administration for chemotherapy tends to widely change in its therapeutic efficiency depending on the injection procedure, such as dose, injection rate, and injection site. The utilization of nanoDDS-embedded injectable hydrogels would mitigate the limitation of instant intratumoral administration. Hydrogel is composed of a network of crosslinked polymers entrapping a large volume of water inside its structure and is frequently explored as a biocompatible drug delivery platform [126]. Hydrogel helps to control the release profile of embedded nanoDDSs in a sustained way, thereby minimizing the washout of nanoDDSs and the consequent systemic toxicity [127]. W. Shen et al. proposed a micelle–hydrogel transition system that enabled the sustained release of both hydrophilic and hydrophobic drugs (Figure 1A,B) [104]. Thermosensitive micelles conjugated with cisplatin and loaded with paclitaxel were intratumorally administered, and the micelles were subsequently transitioned to hydrogel at body temperature, releasing 75% cisplatin over 40 days and 75% paclitaxel over 75 days (Figure 1C). The co-delivery of hydrophilic and hydrophobic drugs was successfully achieved and the sol–gel transition of the micelles promoted their sustained release. This approach exhibited improved anticancer efficacy with reduced side effects against SKOV-3 ovarian cancer xenograft mouse models (Figure 1D). In another study by H. Zhao et al., thermosensitive nanogels composed of poly(styrenesulfonate-b-N-isopropylacrylamide-b-styrenesulfonate) (PNS) triblock copolymers were employed for the intratumoral delivery of Pd(II) ions [105]. PNS was crosslinked by Pd(II) ions to form nanogels (Pd-PNS), and the Pd-PNSs spontaneously assembled into hydrogels at the body temperature. Pd(II) ions were slowly released from Pd-PNS hydrogels, ~70% within 5 days. The intratumoral administration of Pd-PNSs showed an enhanced therapeutic effect on platinum-resistant tumor models.

Microneedles, composed of patches with arrayed small needles, can further offer another option for the sustained intratumoral administration of nanoDDSs. They are minimally invasive when penetrating the primary skin barrier and efficiently facilitate nanoDDS delivery beneath the skin through passive diffusion without inducing irritation to blood vessels or nerves [128,129]. The drug release profile of microneedles can be precisely controlled through the modification of their composition and structure [130,131]. The use of microneedles for the intratumoral administration of chemodrug-encapsulated nanoDDSs ensures their uniform distribution within the tumor tissue and high targeting efficiency. X. Lan et al. loaded cisplatin-encapsulated lipid-coated nanoparticles onto tumor-targeting microneedles [106] (Figure 1E). Through in vitro experiments, a 60% release of cisplatin from the nanoparticles for 72 h was revealed. When cisplatin-encapsulated nanoparticles were intratumorally administered using microneedles, a significant tumor growth inhibition was observed in head and neck squamous cell carcinoma (HNSCC) xenograft Balb/c mouse models, compared to their intravenous or direct intratumoral administration (Figure 1F,G). Furthermore, the microneedle did not exhibit any long-term toxicity and no cisplatin leaked out into the blood either. Overall, the intratumoral administration of nanoDDSs was demonstrated to offer unique advantages in enhancing the efficacy of chemotherapy. The limitations in chemotherapy aroused from the small molecular weights of chemodrugs, such as poor pharmacokinetic properties, low bioavailability, and rapid clearance from tumor tissues, were successfully overcome by intratumoral nanoDDS administration. By utilizing different administration methods depending on the types of nanoDDSs and target tumors, the drug release behavior and intratumoral distribution of nanoDDSs can be adequately adjusted, advancing the field of localized chemotherapy.

### 3.2. Photothermal Therapy (PTT)

Hyperthermia is a therapeutic approach involving the application of heat generated by various energy sources such as lasers, ultrasounds, and microwaves [132,133,134,135,136]. Among these options, lasers are the most preferred energy sources for hyperthermic therapy due to their ability to precisely focus light on the target tumor site with minimal energy loss. Traditional PTT relies on light to directly increase the temperature inside the tumor tissue, which is available only for superficial skin tumors due to poor tissue selectivity and limited penetration depth [137]. In the meantime, the application of metal nanoparticles in PTT has significantly enhanced its therapeutic effectiveness. Gold nanoparticles have gathered the most attention in this field due to their fully established surface modification methods and tunable light absorption properties. When gold nanoparticles are irradiated with light, they exhibit surface plasmon resonance, converting the light energy into heat through non-radiative processes involving electron–phonon interactions. This phenomenon enables gold nanoparticles to be utilized in PTT, inducing cell necrosis or apoptosis in tumors [138]. Gold nanoparticles can be engineered to have different morphologies, such as nanospheres, nanoshells, nanostars, or nanorods [107,139,140,141]. Among them, nanorods are the most commonly employed gold nanoparticles in animal models of PTT since their light absorption wavelength can be conveniently adjusted by controlling their aspect ratios [142].

The intratumoral accumulation of gold nanoparticles is of great importance in PTT as it regulates the heat generation efficiency inside tumor tissues. Several modification methods of gold nanoparticles such as PEGylation, stem-cell-mediated delivery, membrane mimetics, and liposomal formulations have been investigated to overcome their low tumor-targeting efficiency and severe accumulation in the liver and kidneys [134,143,144,145]. However, the systemic administration of gold nanoparticles is too inefficient to achieve their intratumoral accumulation with a sufficient concentration for PTT, wherein intratumoral administration can be a viable alternative. D. K. Yi et al. reported engineered gold nanorods surface-modified with matrix metalloproteinase (MMP)-cleavable Cy5.5 fluorescent dyes [107]. The nanorods were designed to simultaneously implement the visualization of MMP activity and cancer therapy. As the nanorods were intratumorally administered to SCC-7 tumor xenograft mouse models, maximum intratumoral fluorescence intensity was observed after 60 min and the temperature of tumor tissues increased to 45 °C within 4 min upon irradiation with a 671 nm laser. The heat generated by nanorod administration with near-infrared (NIR) irradiation efficiently induced the apoptosis of tumor tissues, confirmed via histological analysis. In another study by Y. Zhang et al., gold nanoparticles capable of aggregation in response to the acidic tumor microenvironment were developed by modifying their surfaces with two peptide pairs that bind together in acidic conditions [108]. The aggregation of gold nanoparticles was confirmed in both in vitro and in situ experiments, which led to a substantial increase in their light absorbance and effective heat generation. After the intratumoral administration, immediately aggregated gold nanoparticles sharply increased the temperature inside the tumor tissues to 70 °C. The aggregated gold nanoparticles in tumor tissues could be also observed via computed tomography (CT), determining their theranostic performance.

**Figure 2:**
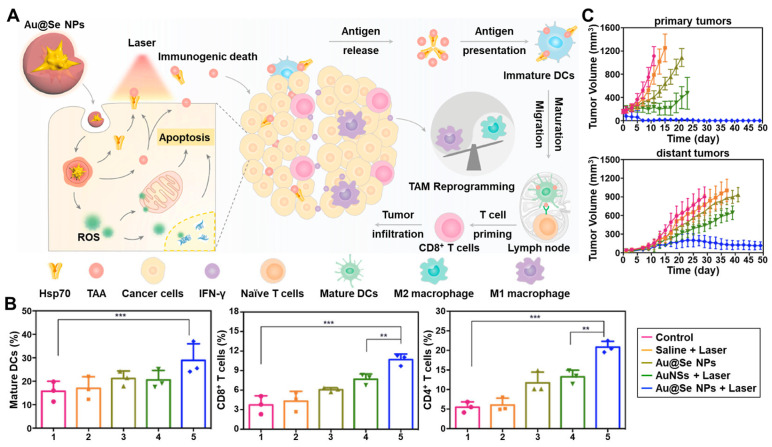
Immune activation by PTT with intratumorally administered nanoDDSs. (**A**) The intratumoral administration of Au@Se NPs and NIR irradiation simultaneously induced the ICD by PTT and dendritic cell activation, enhancing antitumor immunity. (**B**) The maturation of dendritic cells and activation of CD8+ and CD4+ T cells were demonstrated after PTT with intratumorally administered Au@Se NPs. ** *p* < 0.01, *** *p* < 0.001. (**C**) The treatment with Au@Se NPs successfully inhibited the growth of both primary and distant tumors. Reproduced with permission [109]. Copyright 2020, Elsevier.

In several studies, it was revealed that PTT can elicit inflammatory responses by inducing both the apoptosis and necrosis of cancer cells [146]. PTT with high-energy doses is reported to cause cell necrosis, while apoptotic behaviors majorly appear with moderate PTT slightly above body temperature [147,148]. At specific temperatures below 50 °C, PTT can mediate the induction of immunogenic cell death (ICD) which activates antitumor immunity [109,149,150,151]. In particular, PTT-treated cancer cells express upregulated damage-associated molecular patterns (DAMPs) such as heat shock protein 70 (HSP70), facilitate the maturation of dendritic cells, and subsequently promote the infiltration of effector T cells into tumor tissues. J. Wang et al. reported that PTT with gold–selenium core–shell nanoparticles (Au@Se NPs) could convert a “cold tumor” characterized by the absence of effector T cells into a “hot tumor” (Figure 2A) [109]. PTT using intratumorally administered Au@Se NPs was demonstrated to induce ICD and activation of dendritic cells at temperatures around 50 °C, which encouraged the infiltration of CD4^+^ and CD8^+^ T cells into tumor tissues in animal experiments (Figure 2B). Additionally, elevated levels of heat shock protein 70 (HSP70) and immune cytokines within tumor tissues were observed. The activated antitumor immune responses by localized PTT also successfully suppressed remote tumor growth (Figure 2C). This study employed low doses of Au@Se NPs via intratumoral administration to boost ICD at a moderate temperature compared to conventional photothermal therapy. In general, high and exclusive intratumoral accumulation of nanoDDSs was of great importance for PTT since it required high concentrations of nanoparticles for enough heat generation to kill cancer cells, and those nanoparticles for PTT were not biodegradable in most cases [152]. Therefore, intratumoral administration would be a major administration route for photothermal nanoparticles, promoting their tumor-specific localization.

### 3.3. Photodynamic Therapy (PDT)

PDT is a therapeutic modality to kill cancer cells with reactive oxygen species (ROSs) generated by photosensitizers (PSs) and light irradiation. When PSs absorb light of a specific wavelength, they are excited from the ground to a singlet or triplet state, and the excited energy is immediately transferred to adjacent oxygen molecules, resulting in the generation of ROSs such as superoxide radicals and hydroxyl radicals [153]. PDT can be classified into type I and II, wherein type I PDT is accompanied by the intermediation of energy transfer by biomolecules such as thymidine, guanine, tyrosine, and tryptophan to convert oxygens into radicals [154]. In type II PDT, the excited energy is directly transferred from PSs to oxygens to produce singlet oxygen [155]. Diverse PSs composed of either organic or inorganic substances which exhibit different characteristics have been extensively explored for PDT. Organic PSs such as 5-ALA, Ce6, and verteporfin are known to have higher biocompatibility than inorganic PSs, while inorganic PSs including silica, gold, silver, platinum, and iron oxide exhibit higher quantum yields [156]. PDT is only effective in the presence of both PSs and light irradiation, which endows it with a spatio-specific property.

Despite its powerful therapeutic efficacy through ROS generation, PDT has some critical limitations in its application concerning light irradiation [157]. PDT is not a good option for the treatment of deep tumor tissue because light has a short penetration depth. What worsens the deep penetration issue is that most types of PSs are typically more sensitive to ultraviolet (UV) light for which tissue penetration is poor compared to visible (VIS) or NIR light [158]. NIR light is used for PDT considering the tissue penetration depth, but it cannot fully activate the PSs [159,160]. In addition, the undesirable distribution of PSs to normal tissues causes off-target toxicity, especially to the skin and eyes which are frequently exposed to natural light [161]. The poor pharmacokinetic property of PSs due to their high hydrophobicity is another hurdle in PDT application [162]. Moreover, the inhomogeneous intratumoral distribution of PSs results in their localized therapeutic effects in limited tumor regions due to the short half-life of ROSs, stimulating the resistance of tumor tissues against PDT [163]. Therefore, it is essential to enhance the ROS-generating efficiency of PSs under NIR irradiation, specifically to regulate their biodistribution and uniformly deliver them throughout the tumor tissues to secure effective and safe PDT.

**Figure 3:**
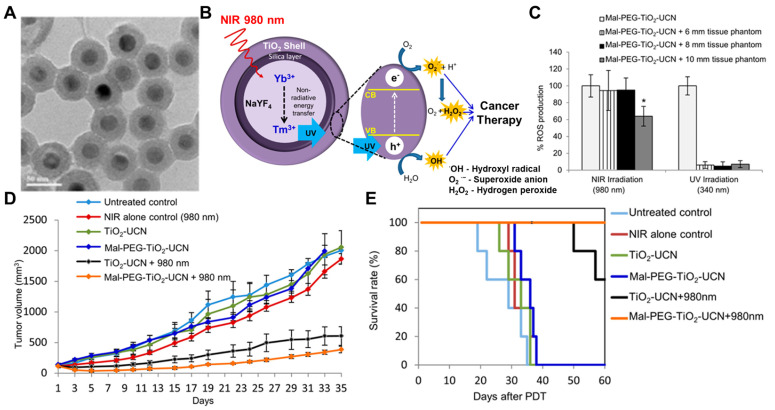
PDT with intratumorally administered Mal-PEG-TiO_2_-UCN nanoparticles. (**A**) A TEM image of synthesized Mal-PEG-TiO_2_-UCNs was obtained. (**B**) The UCNs shifted the wavelength of irradiated light to 450~475 nm, and the TiO_2_ shell absorbed the shifted light to produce ROSs. (**C**) The Mal-PEG-TiO_2_-UCNs effectively generated ROSs even inside the deep tissue phantoms. * *p* < 0.05. (**D**) The intratumoral administration of Mal-PEG-TiO_2_-UCN with 980 nm light irradiation significantly suppressed tumor growth (**E**) without causing any notable side effects. Reproduced with permission [110]. Copyright 2015, ACS Publications.

The intratumoral administration of nanoDDSs can be an advantageous solution for PDT as it effectively adjusts the inferior pharmacokinetic properties of PSs and enhances their tumor-exclusive accumulation. S. S. Lucky et al. proposed the intratumoral administration of titania-coated upconversion nanoparticles (UCN) to improve PDT efficacy [110]. The UCNs composed of NaYF_4_:Yb,Tm were coated with TiO_2_, and maleimide-PEG-silanes (Mal-PEGs) were further conjugated on the surface to obtain Mal-PEG-TiO_2_-UCNs with sizes of 37.5 nm (Figure 3A). TiO_2_ is a powerful inorganic PS with a high quantum yield but is sensitized by UV light only. Since NIR can penetrate tissues more deeply than UV or VIS, UCNs were adopted to absorb light of longer wavelengths (980 nm), upconvert the absorbed light to shorter wavelengths (450~475 nm), and transfer the light to TiO_2_ shells (Figure 3B). The prepared Mal-PEG-TiO_2_-UCNs efficiently generated ROSs even inside the deep tissue phantom under NIR irradiation (Figure 3C). When intratumorally administered, the Mal-PEG-TiO_2_-UCNs significantly inhibited tumor growth with NIR radiation, wherein the tumor volume was 4-fold smaller than the control on day 30 (Figure 3D). All Mal-PEG-TiO_2_-UCN-treated mice survived until the end of the test, and no significant side toxicity was observed (Figure 3E). Similarly, C. Wang et al. developed the organic PS-conjugated UCN (UCN-Ce6) to improve the tissue penetration depth of light [111]. Notably, the PDT efficacy of intratumorally administered UCN-Ce6 was measured against the 4T1 murine breast cancer xenograft mouse models further covered with an 8 mm pork slice. UCN-Ce6 exhibited significant tumor growth inhibition under 980 nm laser irradiation even if the tumor was covered with pork slices, demonstrating the leverage of UCN and NIR application in PDT.

**Figure 4:**
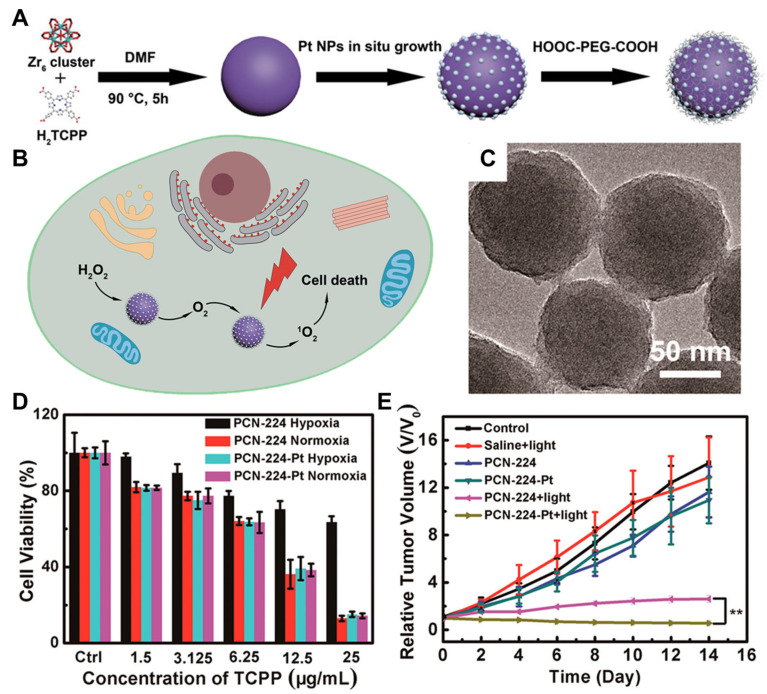
Hypoxia alleviation and PDT by PCM-224-Pt. (**A**) PCM-224-Pt was synthesized via the in situ growth of Pt nanozymes on the PCN-224 MOFs. (**B**) Pt nanozyme produced oxygen from H_2_O_2_, and the oxygen was converted into ROSs by PCN-224 inside cancer cells. (**C**) The synthesized PCM-224-Pt was observed with TEM. (**D**) PCM-224-Pt exhibited high toxicity in both hypoxic and normoxic conditions, whereas the cytotoxicity of PCN-224 was diminished in hypoxia. (**E**) When PCN-224-Pt was intratumorally administered, it greatly promoted tumor regression by alleviating tumor hypoxia and enhancing the effect of PDT. ** *p* < 0.01. Reproduced with permission [112]. Copyright 2018, ACS Publications.

Solid tumors are usually in a hypoxic environment, which diminishes the therapeutic efficacy because the oxygen source inside them required for PDT is insufficient. Therefore, several studies on the external oxygen supply inside tumor tissue were carried out. Y. Zhang et al. designed a metal–organic framework (MOF) system, called Pt nanozymes-decorated porous coordination network-224 (PCN-224-Pt), to achieve both oxygen generation and PDT (Figure 4A) [112]. The Pt nanozymes embedded in the MOF were supposed to catalyze the production of O_2_ from H_2_O_2_ overexpressed inside the tumor tissues, and the generated O_2_ was further converted into ROSs by PCN-224 (Figure 4B). PCN-224 had a diameter of about 90 nm, and Pt NPs deposited on PCN-224 homogenously with a 2 nm thickness (Figure 4C). A cytotoxicity test of PCN-224 and PCN-224-Pt was performed under light irradiation in hypoxia or normoxia, wherein PCN-224-Pt-treated cancer cells presented lower viability in hypoxic conditions compared to PCN-224-treated ones (Figure 4D). In animal tests with H22 tumor xenograft mouse models, the intratumoral administration of PCN-224-Pt thoroughly alleviated the hypoxic condition inside tumor tissues, followed by the improved therapeutic efficacy of PDT (Figure 4E). The PCN-224-treated group showed limited PDT efficacy compared to the PCN-224-Pt-treated group, indicating that tumor-hypoxia-relieving therapy maximizes PDT efficacy in hypoxic solid tumors. In summary, intratumoral nanoDDS administration was demonstrated to be a useful method for PDT since it can modulate the poor pharmacokinetic properties of PSs, reduce the photo-irritation by the undesirable PS biodistribution, and co-deliver the oxygen-source-generating agents as well.

### 3.4. Radiation Therapy (RT)

RT is a treatment method guided by the radiation of high-energy photon beams, which can be divided into external beam radiotherapy (EBRT) and internal radioisotope therapy (RIT) [164]. EBRT is typically performed through the external irradiation of high-energy X-rays, γ-rays, or electron beams, while RIT is carried out by administering radioactive isotopes (RIs) that emit β-particles or γ-rays inside tumor tissues [165]. High-Z radiosensitizers (RSs) such as Au, Ag, Gd, Bi_2_S_3_, and W_x_O_y_ are additionally administered to enhance EBRT, and α-particles, β-particles, and Auger particles such as ^225^Ac, ^211^At, ^213^Bi, ^131^I, ^90^Y, ^64^Cu, ^67^Ga, and ^188^Re are used for RIT [166]. Since both RSs and RIs are extremely toxic and some compounds such as ^131^I, ^223^Ra, and tirapazamine exhibit undesirably long half-lives in the human body, it is important for RT to use appropriate doses of RSs and RIs and minimize their unwanted distribution to normal tissues. In several studies, the application of nanoDDSs in RT showed moderate accomplishments with improved tumor-targeting delivery of RSs or RIs, which were not sufficient due to their extraordinary toxicity.

The intratumoral administration of RS- or RI-encapsulated nanoDDSs notably enhances the RT efficiency while greatly reducing the off-target toxicity. In addition, intratumoral administration significantly reduces their doses compared to systemic administration, leading to further alleviation of their side effects. V. M. Petriev et al. demonstrated that the intratumoral administration of ^188^Re-conjugated silicon nanoparticles (Si*NPs-PEG-^188^Re) for RIT remarkably increased their tumor accumulation and retention compared to their intravenous administration [113]. The Si*NPs were coated with PEGs to evade possible immune responses and ^188^Re was coordinately conjugated to PEGs to avoid the release of free ^188^Re from tumor tissues. The synthesized Si*NPs-PEG-^188^Re had a spherical shape with a size of 25 nm. When Si*NPs-PEG-^188^Re was administered intravenously and intratumorally to RS-1 tumor xenograft Wistar rats and its biodistribution was monitored, the intravenously administered Si*NPs-PEG-^188^Re was mainly detected in the blood, lungs, and kidneys at 5 min post-injection and then gradually accumulated in the kidneys over time. They were hardly delivered to tumor tissues even after 24 h post-injection, while intratumorally administered Si*NPs-PEG-^188^Re remained inside tumor tissues at a high concentration. The intratumoral concentration of intratumorally administered Si*NPs-PEG-^188^Re was 10-fold higher than that with intravenous administration. Notably, the intratumorally administered Si*NPs-PEG-^188^Re exhibited much-prolonged tumor retention compared to the intratumorally administered free ^188^Re, confirming the beneficial effect of nanoDDS on intratumoral administration. The rats intratumorally administered with 37 and 74 MBq of Si*NPs-PEG-^188^Re showed survival rates of 50 and 72% on day 30, respectively, whereas none in the control groups survived.

Hypoxic solid tumors were known to exhibit strong resistance to ionizing radiation due to reduced ROS generation, upregulated DNA repair pathways, and stem-cell-like characteristics [167]. Therefore, similarly to PDT, relief of the hypoxic conditions and focusing the X-ray radiation energy on the tumor would increase the therapeutic efficacy of RT. F. Gong et al. suggested PEGylated core–shell tantalum oxide@manganese dioxide nanoparticles (TaOx@MnO_2_-PEG) with sizes of 100 nm to alleviate tumor hypoxia and achieve more efficient RT [114]. The MnO_2_ shell was intended to induce the decomposition of H_2_O_2_ into oxygen, and TaOx was for the RT. In the cellular assay, TaOx@MnO_2_-PEG imposed stronger DNA damage to 4T1 cancer cells compared to bare TaOx nanoparticles, which was attributed to the oxygen generation by the MnO_2_ shells. The intratumoral administration of TaOx@MnO_2_-PEG dramatically decreased the hypoxic area inside the tumor tissue, resulting in great regression in the tumor volume compared to TaOx administration. Collectively, intratumoral administration of TaOx@MnO_2_-PEG with X-ray irradiation successfully increased the efficiency of EBRT even against tumors with highly resistant hypoxic conditions. Accordingly, the intratumoral nanoDDS administration was proven to promote the tumor-specific localization of RIs or RSs, alleviate the exposure of normal tissues to RT, which is a critical problem of RT, and reinforce its therapeutic potency through the simultaneous delivery of RT-enhancement agents.

### 3.5. Combination Therapy

Combination therapy is the application of multiple therapeutic modalities which can complementarily or synergistically enhance anticancer efficacy with different modes of action (MOAs). Since tumor tissues are often highly resistant to a single therapeutic approach, combination therapy is universally implemented to compensate for the drawbacks of each therapeutic approach [168]. Various matches of therapeutic agents are employed for cancer combination therapy, which can be a simple pair of different anticancer drugs (doxorubicin and paclitaxel, cisplatin and gemcitabine, etc.) or a pair of different therapeutic modalities (chemotherapy and PDT, chemotherapy and RT, etc.) [169,170,171]. Recently, one of the clinical fields in which combination therapy has been most actively adopted is cancer immunotherapy [172]. Cancer immunotherapy is perceived as a potent approach to promote the total remission of tumors and prevent their recurrence by activating the antitumor responses of native immune systems [173]. However, it is difficult to completely activate antitumor immunity with only one immunotherapeutic modality due to deeply immunosuppressive TMEs and the high-dimensional complexity of the immune system, thereby a combinatorial application of treatments with different immune-activating pathways is eagerly awaited [174,175,176].

**Figure 5:**
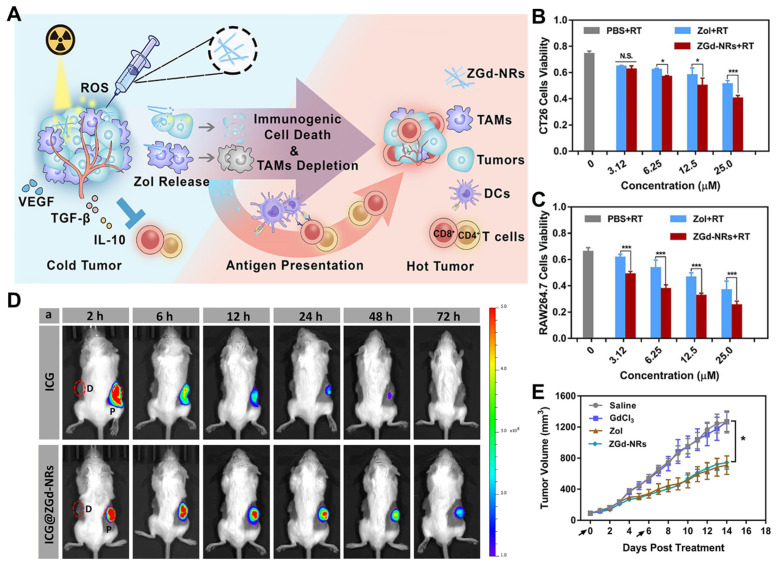
Intratumoral-administration-mediated combined cancer immunotherapy. (**A**) The intratumorally administered ZGd-NRs for combination therapy were supposed to release Zol and Gd^3+^, which depletes TAMs and induces RT-based ICD, respectively, leading to antitumor immunity activation. ZGd-NRs exhibited significant toxicity to both (**B**) cancer cells and (**C**) TAMs. (**D**) The intratumorally administered ZGd-NRs remained inside tumor tissues for a much longer time than the free ICGs (red dot circle and D: distant tumors, P: primary tumors). (**E**) The intratumoral administration of ZGd-NRs showed higher anticancer efficacy compared to the administration of Zol or Gd^3+^ alone, demonstrating the effectiveness of combination therapy. N.S. not significant, * *p* < 0.05, *** *p* < 0.001. Reproduced with permission [115]. Copyright 2017, ACS Publications.

In combination therapy, it is necessary to consider the different pharmacokinetic properties of administered therapeutic agents and secure their sufficient intratumoral accumulation to elicit their synergistic effects. NanoDDSs encapsulated with multiple therapeutic agents have been reported to facilitate their synchronized delivery for combination therapy by modulating their pharmacokinetic properties [177,178]. From this point of view, the intratumoral administration of nanoDDSs is one of the most attractive approaches for combination therapy. Z. Huang et al. investigated zoledronic acid (Zol)-gadolinium (Gd^3+^)-coordinated polymeric nanorods (ZGd-NRs) for intratumoral-administration-mediated combined radio-immunotherapy [115]. ZGd-NRs were synthesized via the self-assembly of Zol and Gd^3+^, which are immunomodulating and RT agents, respectively. They were expected to release both free Zol and Gd^3+^ inside tumor tissues, wherein Zol exhausts tumor-associated macrophages (TAMs) by inhibiting their essential metabolic pathways and Gd^3+^ induces the RT-mediated ICD (Figure 5A). The synthesized ZGd-NRs had a diameter of 20 nm and a height of 200 nm. In the cellular assay, ZGd-NRs exerted cytotoxic effects on both CT26 cancer cells and RAW264.7 macrophages (Figure 5B,C). When monitoring the biodistribution of indocyanine green (ICG)-dye-labeled ZGd-NRs after their intratumoral administration, ZGd-NRs were retained inside tumor tissues for up to 72 h, while free ICG was rapidly washed out within 6 h (Figure 5D). In vivo assessment with CT26 tumor xenograft mouse models demonstrated that the intratumoral administration of ZGd-NRs exhibited higher antitumor efficacy compared to the administration of Zol or Gd^3+^ alone (Figure 5E). Moreover, ZGd-NRs strongly activated antitumor immunity by inducing both TAM depletion and ICD, resulting in the growth inhibition of distant tumors. No significant side effect was observed during the ZGd-NR treatment. Therefore, the effectiveness of immunomodulation–radiosensitization combined therapy mediated by intratumoral nanoDDS administration was fully determined for its application to cancer immunotherapy. Combinatorial cancer immunotherapy was also conducted via the intratumoral administration of immunoadjuvants and PTT agents. L. Guo et al. synthesized the cytosine–guanine (CpG)-loaded chitosan-coated hollow copper sulfide (CuS) nanoparticles (HCuSNPs-CpG) which were intended to release CpGs and CuS crystals under NIR irradiation and separately reassemble to improve their tumor retention [116]. The CpGs and CuS crystals played roles in specifically activating toll-like receptor 9 (TLR-9)-presenting dendritic cells and inducing PTT-mediated ICD, respectively. The biodistribution analysis of intratumorally administered HCuSNPs-CpGs showed their prolonged tumor retention for 24 h. Intratumorally administered HCuSNPs-CpGs were inactively maintained before the NIR excitation and exhibited a potent antitumor efficacy with NIR irradiation which was stronger than that with the separate treatment with HCuSNPs and CpGs. The synergistic activity of CpGs and CuS crystals successfully activated the systemic anticancer immune responses, promoting the growth inhibition of distant tumors. The intratumoral administration of nanoDDSs was determined to be effective for the spontaneous delivery of two or more therapeutic agents with different pharmacokinetic properties, enhancing their combinational therapeutic effects.

### 3.6. Other Therapeutic Modalities

Small interfering RNA (siRNA) is a class of RNA molecules that plays a crucial role in gene silencing and the post-transcriptional regulation of gene expression. siRNA molecules are negatively charged and have double-stranded structures around 20~25 base pairs in length. Due to the poor physiological stability of naked siRNA, its systemic administration is highly challenging, and the use of siRNA carriers is essential for its tumor delivery [179,180,181]. Both viral and non-viral carriers have been developed to facilitate the delivery of siRNA, wherein positively charged lipids and polymers are the primary option for non-viral carriers as they can form stable electrostatic complexes with negatively charged siRNAs [182,183,184]. In addition, intratumoral administration is the major route for siRNA delivery since the bioavailability of its systemic delivery is frustratingly low compared to other therapeutic agents, despite using nanoDDSs [185]. S. M. Noh et al. developed PEGylated poly-L-arginine derivatives of chitosan (PEG-CS-PLR) copolymers for the intratumoral administration of siRNAs [117]. siRNAs could interact with positively charged poly-L-arginine and chitosan in the copolymers and self-assemble into nanoparticles with sizes of 375 nm. The PEG-CS-PLR/siRNA nanoparticles were taken up by cancer cells, delivering free siRNAs intracellularly and silencing the target proteins. The intratumoral administration of PEG-CS-PLR/siRNA nanoparticles significantly reduced the intratumoral expression levels of the target protein, which was about 12-fold lower than that with naked siRNA treatment. Further, H. Na et al. employed mesoporous silica nanoparticles (MSNPs) as siRNA carriers and examined the cancer immunotherapeutic efficacy of their intratumoral administration [118]. The polyethyleneimine (PEI)-PEG copolymer-coated silica nanoparticles with sizes of ~100 nm were synthesized via the sol–gel method, and both STAT3-inhibiting siRNAs and immunoadjuvants (CpGs) were bound to the positively charged PEI coatings to obtain siSTAT3-CpG-NPs. siSTAT3-CpG-NPs were designed for tumor vaccination by knocking down the immunosuppressive pathways and activating antigen-presenting cells (APCs). The intratumoral administration of siSTAT3-CpG-NPs efficiently inhibited the growth of both local and distant tumors by reprogramming the immunosuppressive TMEs.

Proteolysis-targeting chimeras (PROTACs) have recently attracted lots of interest in cancer therapy since they can catalytically regulate targeted oncogenic protein levels using the innate cellular system [186]. PROTACs consist of ligands for the target protein, linker, and an E3 ligase-recruiting moiety. When internalized to cells, PROTACs simultaneously bind to both target proteins and E3 ligases to form ternary complexes, catalyzing the polyubiquitination and proteasomal degradation of target proteins [187]. Although PROTACs have proven their outstanding efficacy at cellular levels, their clinical translation has not succeeded yet due to their inferior pharmacokinetic behavior. The pharmacokinetic property of PROTACs is even worse than that of conventional hydrophobic chemodrugs due to their high molecular weight and hydrophobicity, which leads to their rapid clearance and undesirable biodistribution [188,189]. The nanoDDS formulation of PROTACs and their intratumoral administration would be a practical method to overcome their shortcomings and conveniently exploit their therapeutic effects. Q. He et al. investigated the intratumoral administration of NIR-activatable PROTAC nanocages (UMSNs@phoBET1) [119]. The NIR-activatable bromodomain 4 (BRD4)-degrading PROTACs (phoBET1) were loaded inside the UCNs-based mesoporous silica nanoparticles, which could release free PROTACs under 980 NIR laser irradiation. The released PROTACs induced cancer cell apoptosis by degrading BRD4 and inhibiting the transcription of oncogenes. Intratumorally administered UMSNs@phoBET1 showed effective tumor growth inhibition, a 1.2-fold higher effect than free PROTAC treatment, without causing any side effects or weight loss. Remarkably, the UMSNs@phoBET1 expressed no therapeutic effects without the NIR irradiation. Furthermore, X. Li et al. developed aptamer-based c-Myc-degrading PROTACs (TEPs) and loaded them in cationic liposomes for immuno-combination therapy [120]. c-Myc is a master transcription factor for numerous oncogenic proteins and exhibits a specific affinity for threose nucleic acid (TNA) aptamers. TEPs were synthesized via the conjugation of TNA aptamer-E-box DNA and E3 ligase-recruiting pomalidomide and loaded inside the cationic liposomes to secure better stability. TNA aptamer binds the c-Myc/Max heterodimer, and E-box DNA acts as a bivalent binder. The TEPs inhibited the proliferation of cancer cells by degrading the overexpressed c-Mycs. The intratumorally administered TEP-loaded liposomes significantly suppressed tumor growth with the downregulation of c-Myc in tumor tissues. In summary, the intratumoral nanoDDS administration was extensively applicable for delivering various therapeutic agents with exceptionally poor bioavailability, supporting them to elicit their maximal therapeutic efficacy.

## 4. Clinical Application of Intratumoral Administration

NanoDDSs have undergone extensive preclinical investigations, some of which have progressed into clinical trials. Doxil is a representative nanoDDS that has been granted Food and Drug Administration (FDA) approval for its intravenous administration to treat various cancers [190,191]. When entering clinical studies, the administration route of nanoDDSs should be determined depending on their intended use and target site, wherein intravenous administration is most commonly considered. Currently, there are two different types of nanoDDSs reported to have entered clinical trials. The first clinical trial of intratumoral nanoDDS administration is for Nanotherm^®^, which received approval from the European Medicines Agency (EMA) in 2010 for the treatment of glioblastoma and prostate cancer [192]. Nanotherm^®^ comprises 15 nm-sized paramagnetic iron oxide nanoparticles coated with aminosilane. It is supposed to be intratumorally administered and applied with a magnetic field to induce heat, thereby ablating the tumor [193]. Nanotherm^®^ is undergoing a Phase 2B clinical study in the United States for its intratumoral administration (NCT05010759). The second nanoDDS is NBTXR3/Hensify, a radioenhancer for the treatment of locally advanced soft tissue sarcomas, which obtained the CE Mark in 2019 [194]. Developed and marketed by Nanobiotics, this nanoDDS consists of HfO_2_ cores with sizes of 50 nm surrounded by a negatively charged phosphate coating [195]. When NBTXR3/Hensify is intratumorally administered and subsequently exposed to ionizing radiation, it generates additional electrons which enhance the radiation-induced cell death [196]. NBTXR3/Hensify is the focus of a recent FDA clinical study (NCT02805894) and is being investigated for lung cancer treatment in combination with immunotherapy (NCT03589339) as well.

## 5. Conclusions

In this review, the intratumoral administration of nanoDDSs was covered regarding its advantages over systemic administration, influencing factors on its efficiency, its application to different therapeutic methods, and its current status in clinical trials. The factors affecting the tumor accumulation and distribution of intratumorally administered nanoDDSs were classified and explained as the tumor microenvironment, nanoDDS property, and administration process. This paper was further categorized according to therapeutic areas, and the advantages and experimental results in each area were introduced. The intratumoral administration of nanoDDSs was demonstrated to ensure improved therapeutic outcomes compared to systemic delivery regardless of therapeutic modalities by promoting their tumor-specific accumulation and retention. In particular, intratumoral nanoDDS administration was more effective for the therapeutic modalities whose therapeutic agents have poor pharmacokinetic properties and high systemic toxicity, such as PDT and RT. Although there is still a limitation in the intratumoral administration of nanoDDSs due to the restricted accessibility to tumor tissues located deep inside the body, its exceptional tumor-specific targetability and superior side-effect-mitigating performance make it a highly attractive option for cancer therapy. Moreover, intratumoral nanoDDS administration has been broadly adopted for cancer immunotherapy, which is the recent mainstream in cancer therapy. Intratumoral-administration-mediated cancer immunotherapy is effective at treating not only primary tumors but also distant ones by activating anticancer immunity, which also solves the drawback of intratumoral administration. Accompanied by the continuous advancement in the development of intratumoral drug-releasing interventional devices and implants which can facilitate the tumor approach, intratumoral nanoDDS administration is expected to be a strong alternative to conventional systemic drug delivery.

## Figures and Tables

**Table 1:** Application of intratumoral nanoDDS administration to different therapeutic modalities and its results.

Therapeutic Modality	NanoDDS Composition	Therapeutic Agent	NanoDDS Size/Charge	Results	Ref.
Chemotherapy	PLGA nanoparticle	Doxorubicin	128.0 ± 3.9 nm /11.8 ± 3.4 mV	Sustained drug release for 14 days, minimal toxicity, anticancer immunity activation	[103]
	PEG-PLGA thermosensitive micelle	Cisplatin, paclitaxel	~17 nm/N.A. ^1^	Sustained drug release for 75 days, minimal toxicity, synergistic anticancer effect	[104]
	Thermosensitive PNS nanogel	Pd(II)	158.4 ± 6.5 nm /−4.4 ± 1.2 mV	Sustained drug release for 5 days, long-term intratumoral retention, effect on Pt-resistant tumors	[105]
	Lipid nanoparticle-embedded microneedle	Cisplatin	63.6 ± 15.2 nm /N.A.	Sustained nanoparticle release, reduced systemic toxicity, enhanced anticancer effect over systemic administration	[106]
Photothermal therapy	MMP-cleavable peptide-conjugated gold nanorod	Gold nanorod	~40 × 20 nm /N.A.	Efficient photothermal effect, theranostic property	[107]
	Peptide-conjugated gold nanoparticle	Gold nanoparticle	~40 nm /−23.6 ± 3.12 mV	Efficient photothermal effect by nanoparticle aggregation, improved intratumoral retention, theranostic property	[108]
	Gold nanostar-embedded selenium nanoparticle	Selenium, gold nanostar	~122 nm /−24.2 mV	Synergistic anticancer effect, efficient therapeutic efficacy with low dose, anticancer immunity activation	[109]
Photodynamic therapy	Maleimide-PEG-coated TiO_2_-upconversion nanoparticle	TiO_2_-upconversion nanoparticle	~50 nm /−15.3 ± 5.3	Enhanced photodynamic effect with NIR light, improved intratumoral retention, repeatable PDT	[110]
	PEGylated and Ce6-conjugated upconversion nanoparticle	Ce6-conjugated upconversion nanoparticle	~30 nm/N.A.	Enhanced photodynamic effect with NIR light, uniform intratumoral distribution, improved therapeutic efficacy	[111]
	Pt nanoparticle-incorporated metal–organic framework	H_2_TCPP, Pt nanozyme	~90 nm/N.A.	Enhanced photodynamic effect with oxygen source generation	[112]
Radiation therapy	PEGylated silicon nanoparticle	^188^Re	~25 nm /N.A.	Tumor-specific accumulation and retention compared to systemic delivery, improved radiotherapy	[113]
	PEGylated and TaOx-coated MnO_2_ nanoparticle	TaOx, MnO_2_	~100 nm/N.A.	Enhanced radiation effect with oxygen source generation, theranostic property	[114]
Combination therapy	Zoledronic acid-Gd^3+^-coordinated polymeric nanorod	Zoledronic acid, Gd^3+^	200.1 ± 10.4 nm /−15.43 mV	Synergistic cancer radio-immunotherapy	[115]
	Chitosan-coated hollow CuS nanoparticle	Cytosine-guanine, CuS	~85 nm/N.A.	Improved intratumoral retention, synergistic photo-immunotherapy	[116]
Others	PEGylated and PLR-conjugated chitosan	siRNA	375.1 ± 188.4 nm /23.9 ± 3.6 mV	Stable siRNA complex formation	[117]
	PEI-PEG copolymer-coated silica nanoparticle		106 ± 0.4 nm /6.5 ± 0.4 mV	Synergistic antitumor immunity activation, reduced systemic toxicity	[118]
	Upconversion nanoparticle-based mesoporous silica nanoparticle	PROTAC	~105 nm /−10.0 mV	NIR light-activatable therapeutic effect, reduced systemic toxicity	[119]
	Liposome		~155.0 nm /1.7 mV	Improved therapeutic efficacy	[120]

^1^ N.A.: not available.

## Data Availability

No data was created in this review.
